# Case Report: Kaposiform Hemangioendothelioma With Spinal Involvement

**DOI:** 10.3389/fped.2021.600115

**Published:** 2021-04-12

**Authors:** Tong Qiu, Kaiying Yang, Shiyi Dai, Siyuan Chen, Yi Ji

**Affiliations:** ^1^Division of Oncology, Department of Pediatric Surgery, West China Hospital of Sichuan University, Chengdu, China; ^2^Pediatric Intensive Care Unit, Department of Critical Care Medicine, West China Hospital of Sichuan University, Chengdu, China

**Keywords:** kaposiform hemangioendothelioma, spinal involvement, scoliosis, Kasabach-Merritt phenomenon, decreased range of motion, clinical manifestations

## Abstract

**Introduction:** Kaposiform hemangioendothelioma (KHE) is a rare, locally invasive vascular tumor that mostly appears in infants and adolescents. KHE with spinal involvement is extremely rare. The aim of this study was to review the imaging features, clinical manifestations and treatment of KHE patients with spinal involvement.

**Materials and Methods:** We reviewed patients with KHE who were admitted to Pediatric Surgery of West China Hospital of Sichuan University from April 2014 to August 2020, and the cases were evaluated.

**Results:** Seven patients with spinal involvement were enrolled in the study, including four (57.1%) males and three (42.9%) females. The age at onset ranged from 1.0 day to 4.0 years, with an average of 1.6 years. Five (71.4%) had pain due to bone destruction, three patients (42.9%) had decreased range of motion (ROM), four (57.1%) patients had scoliosis, two (28.6%) patients developed claudication, and three patients (42.9%) presented with a soft tissue mass in the neck of the back. Five patients (71.4%) had the Kasabach-Merritt phenomenon (KMP), with a minimum platelet value of 8 × 10^9^/L. All patients were treated with sirolimus, and showed regression of the lesion and/or normalization of the hematologic parameters.

**Conclusion:** KHE with spinal involvement is difficult to diagnose due to its rarity and variable symptoms, which need to be recognized to start early treatment. The management of KHE with spinal involvement should be performed by a multidisciplinary team. Sirolimus can improve outcomes in patients with KHE with spinal involvement.

## Introduction

KHE is a rare, locally invasive vascular tumor that mostly appears in infants and adolescents. Epidemiological surveys have shown that the incidence of KHE is approximately 0.71/100,000 in children (1). KHE can be accompanied by a severe complication known as the Kasabach-Merritt phenomenon (KMP), which is characterized by thrombocytopenia, hypofibrinogenemia, and clotting factor consumption (2–5). According to the morphology and the clinical manifestations, Ji (6) divided KHE into three types, namely, superficial (which is limited to the skin and subcutaneous soft tissue without the invasion of muscles and bones, chest and abdominal cavity), mixed (which involves both the skin and subcutaneous deep muscles, bones and/or joints) and deep (which has no skin manifestations). Among these three types, the most common is the mixed type, which accounts for 63.0%.

KHE usually tends to have a focal aggression involving one or more body parts, including the limbs, torso, head and neck, without distant metastasis (7). Approximately 88% of KHE manifests as a purple or red hard mass involving the skin with an unclear boundary separating it from the surrounding tissues. The other 12% of KHE manifests as deep lesions with no skin involvement that can affect not only the retroperitoneum but also the deep muscles, bones, joints, and mediastinum (8). Although deep KHE has variable manifestations, cases with multisegmental spinal involvement are rare. In this study, we reviewed the imaging features, clinical manifestations and treatment of KHE patients with spinal involvement with the aim of improving our understanding of this rare disease and our control of aggressive tumor behavior and patient morbidity.

## Methods

KHE patients admitted to the Pediatric Surgery of West China Hospital of Sichuan University from April 2014 to August 2020 were enrolled. The clinical and imaging data of the KHE patients were collected. The inclusion criteria were as follows: (1) the diagnosis of KHE was confirmed by pathological examination; (2) the clinical manifestations, laboratory examination results, and imaging features were in accordance with the diagnostic criteria of KHE when there was no pathological examination result available; and (3) imaging data showed that the lesions involved but were not limited to the spine. The exclusion criteria were as follows: (1) the absence of detailed clinical data and (2) clinical features and pathological findings that did not meet the KHE diagnostic criteria. The protocol was approved by the Institutional Review Board of the university, and all the patients or patients' parents signed an informed consent form.

## Results

### Anatomical Distribution

According to the inclusion and exclusion criteria, a total of 7 KHE patients with spinal involvement were enrolled in the study, including 4 males and 3 females. The age at onset ranged from 1.0 day to 4.0 years, with an average of 1.6 years. A multidisciplinary team, including experts in radiology, pathology, pediatric surgery and pediatric oncology/hematology, participated in the diagnosis and treatment of KHE.

Among these seven patients, two patients (28.6%) had involvement of the spine and paravertebral region, two patients (28.6%) had involvement of not only the vertebrae and paravertebral region but also the ilium, skull, femur or ribs. Three patients (42.9%) had lesions involving the vertebrae with a subcutaneous soft tissue mass in the neck, axilla, back, chest wall and other parts or a mediastinal soft tissue mass. All had local skin manifestations.

### Clinical Features

Of the seven patients with KHE, five (71.4%) had pain due to bone destruction (whether the other two patients had pain was unclear because they were <1 year old). Three patients (42.9%) had decreased range of motion (ROM), and four (57.1%) patients had scoliosis. Two patients developed claudication. One patient suffered respiratory distress, pneumonia, obstructive pulmonary emphysema and pericardial effusion from obstruction of the airway due to the tumor in the neck and mediastinum. Three patient (42.9%) presented with a soft tissue mass in the neck or the back.

The definition of the KMP is a platelet count <100 × 10^9^/L with or without hypoproteinemia and coagulopathy. Five KHE patients (71.4%) had the KMP, with minimum platelet values of 8 × 10^9^/L. They had an average age of 0.9 year. The routine blood, biochemical, coagulation and other test results of the other two patients (28.6%) were in the normal ranges.

### Pathological Findings

**Figure 1:**
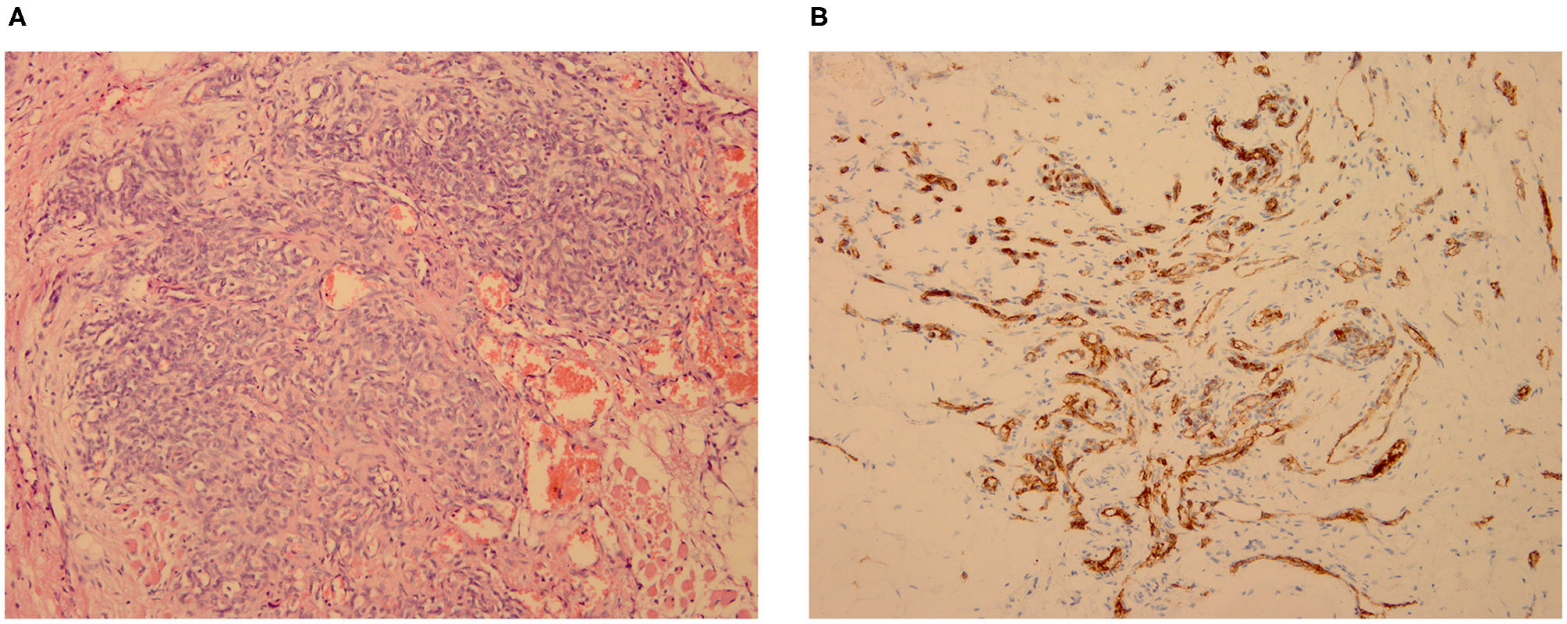
KHE in Patient 2: pathological biopsy of the mediastinal mass, which was located in the deep muscular layer of the left cervical root. (**A**. HE × 200; **B**. CD31 × 200).

Five of the seven patients underwent biopsy of the lesions, and the results were consistent with the pathological changes observed in KHE. The vascular tumors were observed in the fibrous tissue and trabecular bone; they were arranged in a lobulated or nested manner, and the cells in some regions were fusiform (Figure 1A). Immunohistochemical staining showed positivity for CD31 (Figure 1B), CD34, and D2-40 but not for Glut-1 and HHV-8.

### Imaging Findings

The initial imaging examination included X-ray, CT, MRI, ultrasound and whole-body bone scans. X-ray showed bone destruction at the site of the lesion. CT scan showed mixed sclerosis and lytic lesions. Irregular soft tissue masses could be seen near the vertebral body. The MRI scans showed that the lesions had low signal changes on the T1-weighted images and high signal changes on the T2-weighted images. Whole-body bone scans indicated a slight increase in radioactivity distribution in the lateral margin of the diseased vertebral body.

### Treatment and Follow-Up

**Figure 2:**
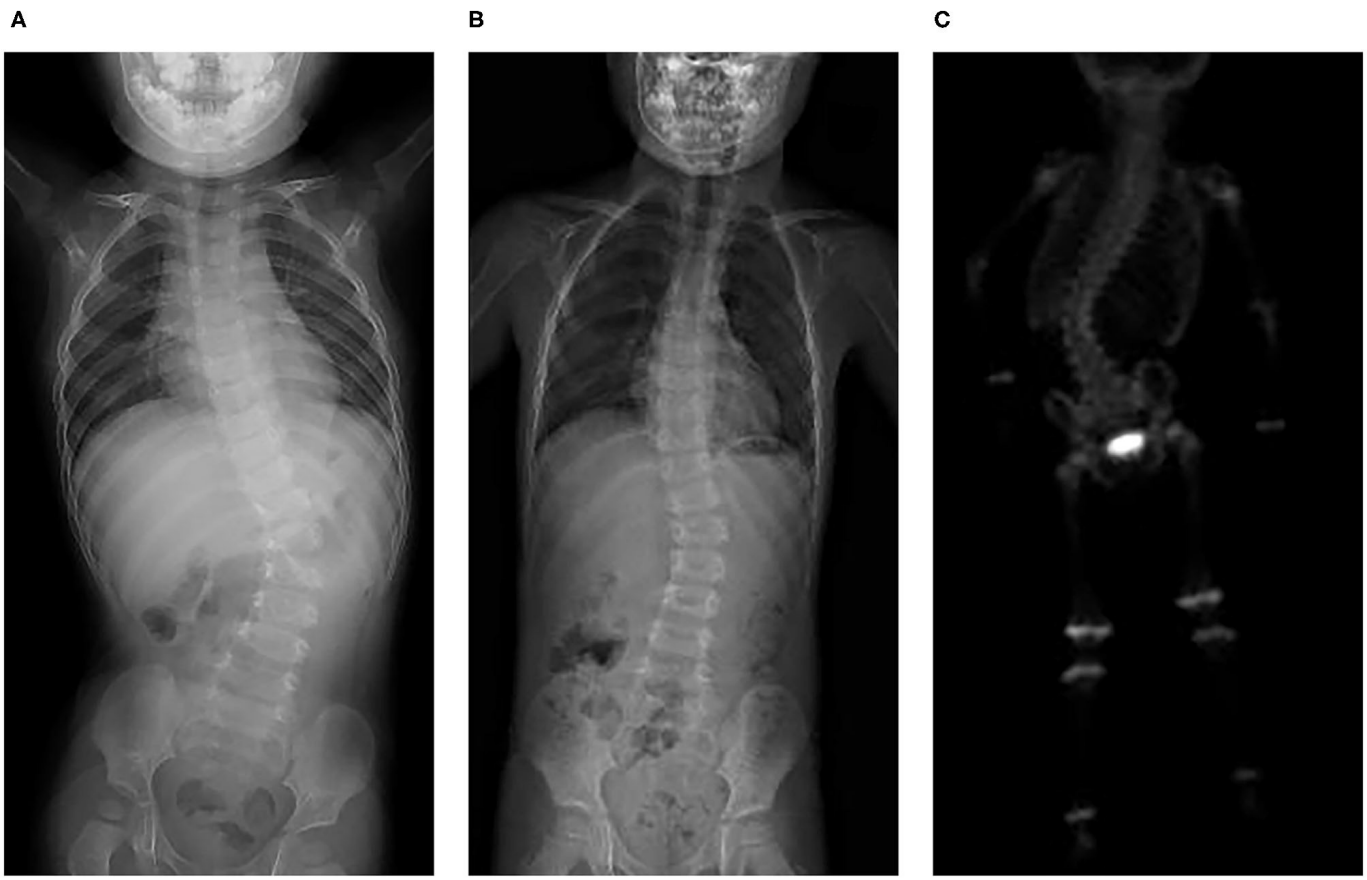
KHE in Patient 2: X-ray showed slight scoliosis **(A)**, and the angle of the scoliosis was reduced after 3 years of treatment and follow-up **(B)**. Three hours after 99 mTc-MDP 10 mCi was injected intravenously, a whole-body bone scan was performed. It also showed scoliosis and slightly increased radioactive distribution on the right edge of the T12-L2 vertebral body **(C)**.

**Figure 3:**
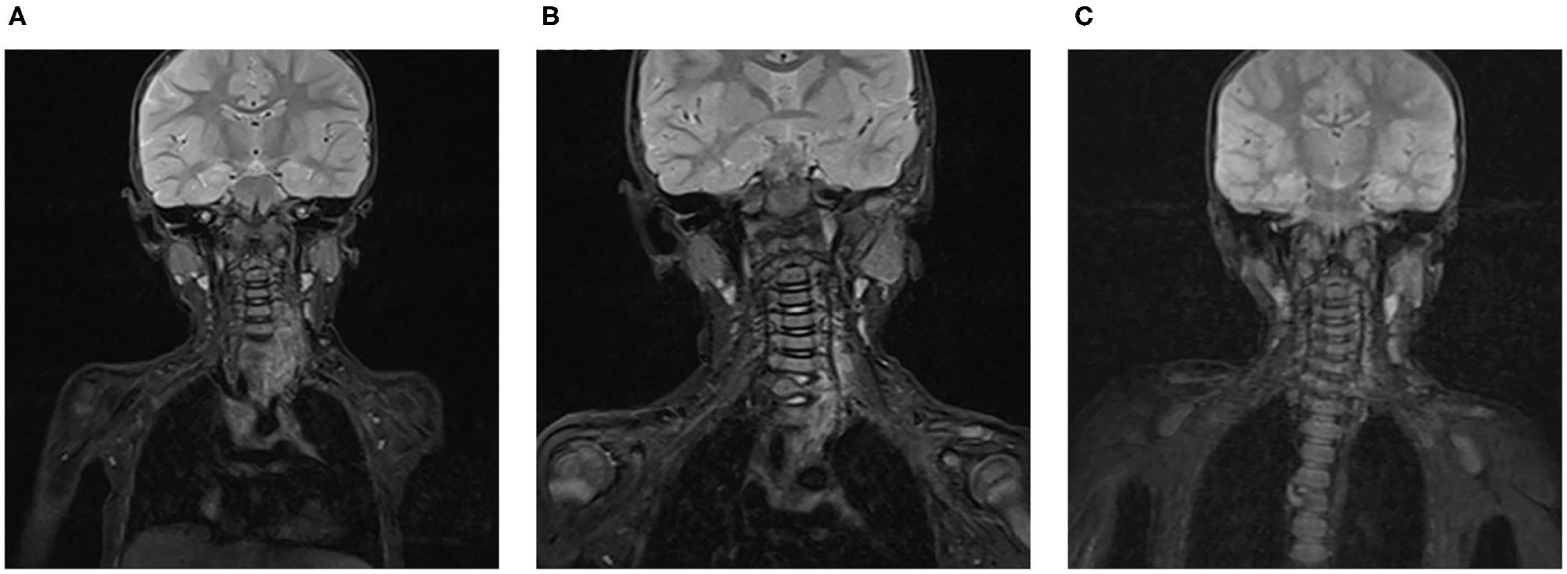
KHE in Patient 3: On MRI, a soft tissue mass in the left root of the neck can be seen before treatment **(A)**. After 6 months **(B)** and 1 year **(C)** of sirolimus treatment, the size of the lesion was gradually reduced.

**Figure 4:**
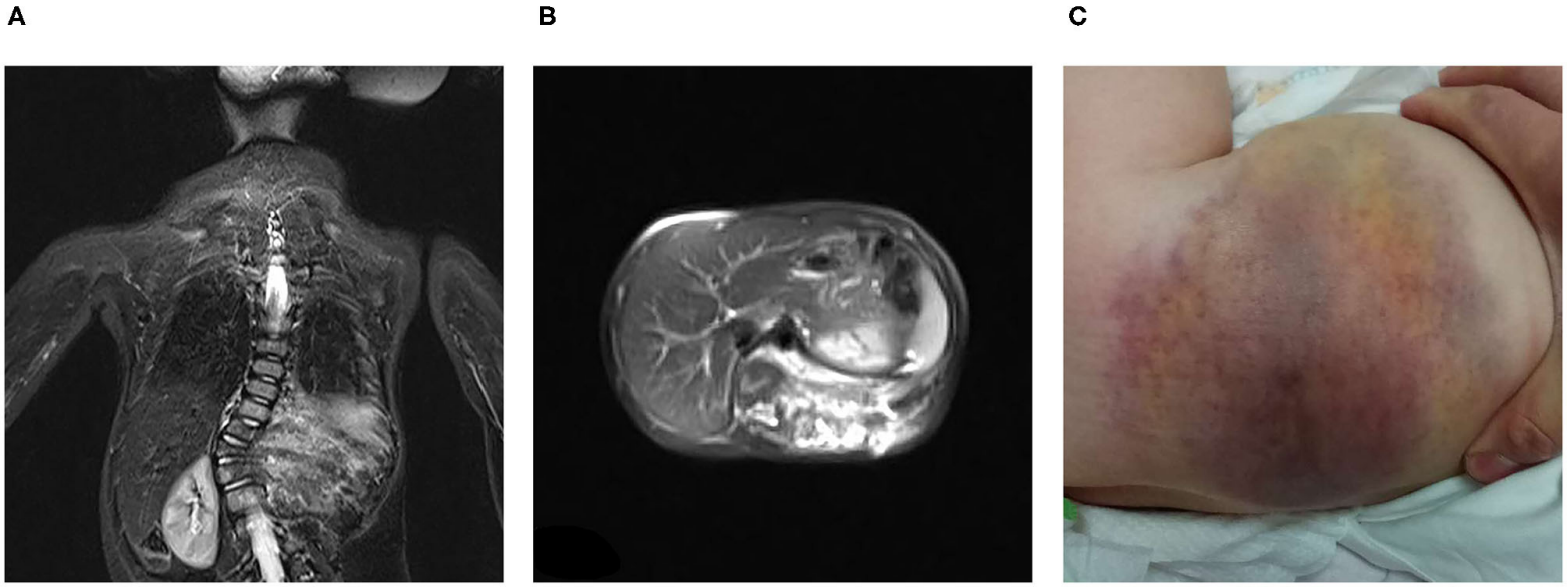
KHE in Patient 6: The MRI showed that large mixed signal mass involved the left lumbar and back, and T7-L4 vertebrae and paraspinal tissue from coronal **(A)** and sagittal position **(B)**, and we can see that the skin on the left waist and back was involved **(C)**.

All seven patients were treated with sirolimus (0.8 mg/m^2^, tid po), and all the patients were improved. Patient 1 was treated with L4-5 vertebral tumor resection biopsy through the posterior lumbar approach with spinal decompression, nerve root release, orthopedic internal fixation and autogenous bone graft fusion. After these treatments, sirolimus therapy was started. MRI showed nearly complete involution of the lesion after 24 months of sirolimus treatment. Patient 2 was admitted to the hospital due to scoliosis (Figure 2). His left back was uplifted and showed a “razor back” deformity. This patient underwent 12th right rib resection through the lateral anterior approach and paravertebral tumor partial biopsy, which indicated a diagnosis of KHE. Then, the patient received oral sirolimus treatment for 3 years, with a significant decrease in the size of lesions of the paravertebral soft tissue during follow-up and a reduction in the Cobb angle of the scoliosis by 38° to 28°. Patient 3 had mediastinal occupancy, accompanied by respiratory distress, obstructive pulmonary emphysema and thrombocytopenia. Mediastinal CT showed multiple lesions of different sizes and node mass shadows of soft tissue in the left neck, mediastinum and left posterior chest wall. Her left main bronchus was significantly narrowed. The body and accessory of the junction between the cervical and thoracic vertebrae, T1 and T6, showed bone destruction (Figure 3). KHE was considered after mediastinal mass biopsy, and sirolimus combined with propranolol was given. This patient was followed up once every 6 months. After 2 years, the sizes of the local lesions were reduced, and her respiratory symptoms were significantly relieved. Patient 4 was admitted to the hospital due to thrombocytopenia and a mass in the left neck. The patient was too young to undergo biopsy. CT showed an irregularly shaped soft tissue mass in the left neck, and there was destruction of multiple bones, including the atlas and axon. We diagnosed the patient with KHE and prescribed oral prednisone and sirolimus, which led to a significant decrease in lesion size and the associated symptoms. Patient 5 went to the hospital because of abnormal walking and the scattered cutaneous markings in the skin. MRI suggested that L3-S1 and paraspinal tissue showed a large range of mixed signal. Sirolimus was used to treat this patient after the paraspinal soft tissue biopsy. Half a year later, MRI showed that the focus was smaller than before and the degree of compensatory scoliosis was alleviated. Patient 6 and patient 7 were treated because of the progressive enlargement masses of the back. After improving the MRI examination, it showed a large mixed signal mass on the left lumbar and back, and T7-L4 vertebrae and paraspinal tissue were involved in patient 6 (Figure 4). The focus was not clearly separated from the adjacent thoracolumbar vertebrae and dorsal muscles, accompanied by bone destruction of thoracolumbar vertebrae, spinal and left thoracic compression with scoliosis deformities. Seven lesions. The lesions of patients 7 were involved left psoas major muscle, left perirenal space and paraspinal tissue, but she did not undergo biopsy because of her younger age without informed consent of her parents. But these two patients were treated with sirolimus orally, and the KMP was quickly improved and platelet value returned to normal. Three months later, MRI showed both of the lesions were smaller. (Table 1).

**Table 1:** Clinical characteristics of patients with KHE involving the spine^*^.

**Cases**	**Sex**	**Age at the onset of KHE**	**Age at the time of the presentation of the KMP**	**Age at the time of the diagnosis of KHE**	**Location**	**PLT (× 10^**9**^/L)**	**FIB (g/L)**	**HGB (g/L)**	**Major signs, symptoms, and/or complications**	**Prior treatment**	**Outcome**
1	Male	4.0 y	-	5.5 y	Lumbar vertebrae, right ilium and femur	359	1.92	151	Pain, decreased ROM, scoliosis, claudication	Propranolol, sirolimus	Improved
2	Male	2.3 y	-	2.5 y	Thoracic and lumbar vertebrae and muscle (paraspinal), rib	266	2.31	113	Pain, decreased ROM, scoliosis	Sirolimus	Improved
3	Female	0.8 y	0.8 y	0.9 y	Neck, axilla, mediastinum, cervical and thoracic vertebrae	21	0.51	88	Respiratory distress, pneumonia, obstructive pulmonary emphysema, pericardial effusion	Sirolimus, propranolol	Improved
4	Male	1.0 d	1.0 d	3.0 d	Neck, cervical vertebrae	22	0.64	179	Neck mass	Corticosteroids, sirolimus	Improved
5	Male	1.8 y	1.8 y	1.9 y	Lumbar and sacral vertebrae and muscle (paraspinal)	8	1.81	114	Scoliosis, decreased ROM, claudication, pelioma, pain	Biopsy, sirolimus	Improved
6	Female	1.4 y	1.4 y	1.4 y	Thoracic and lumbar vertebrae and muscle (paraspinal), back	19	1.74	117	Back mass, scoliosis, pain	Biopsy, sirolimus	Improved
7	Female	0.6 y	0.6 y	0.6 y	Lumbar vertebrae, back	35	0.60	117	Back mass	Sirolimus	Improved

* *PLT, platelets; FIB, fibrinogen; HGB, hemoglobin; ROM, range of motion; y, year; m, month; d, day*.

## Discussion

KHE is a rare, locally invasive borderline tumor that mainly occurs in children. The lesions can involve the skin, with cutaneous manifestations. Refractory thrombocytopenia and coagulation dysfunction can occur when the KMP occurs. A small number of KHE patients have no skin involvement, and lesions involving the spine are extremely rare. Reviewing the cases had been published, we can see that Lisle JW (9) reported a case of KHE without skin changes involving the thoracolumbar spine. The patient was treated with lesion resection. Keskindemirci G (10) reported a 3-year-old boy with extensive involvement of the T11-L5 vertebral body and the paravertebral and retroperitoneal areas. The patient died from intracranial hemorrhage. Zhu Y (11) reported a case involving the thoracic spine with scoliosis. Liu FS (12) reported a 36-year-old adult female with cervical lesions and pain. In all cases, the lesions involving the spine were in multiple segments and were accompanied by vertebral soft tissue mass shadows. In the same vertebrae, tumors can cross the growth plate and simultaneously involve the pedicle and the vertebral body. This shows that KHE has local invasive ability.

When differentiating KHE from other diseases, such as Langerhans cell histiocytosis, neurogenic tumors, spinal tuberculosis, and non-specific infections, early lesion biopsy is recommended to facilitate the earliest possible initiation of treatment. Because of the high level of risk associated with vertebral biopsy, soft tissue biopsies such as those of the paravertebral tissue can be considered.

Idiopathic scoliosis is the most common form of adolescent spinal deformity, and asymmetric growth of the vertebrae is considered to be a possible pathogenic mechanism underlying idiopathic scoliosis (13). In patients with spinal KHE, when the tumor invades the growth plate on one side of the vertebral body, growth is inhibited, causing asymmetric growth of the vertebral body. Eventually, the vertebral body collapses, and scoliosis occurs (14). In addition, pain is caused by tumor stimulation, a large amount of inflammatory cell infiltration and cellulose deposition in the paravertebral soft tissue tumor leading to fibrosis. Local contracture mechanically pulls on the vertebral body and may cause compensatory scoliosis (15, 16).

Currently, treatment experience of spinal KHE is lacking. Vincristine was previously considered a first-line medical therapy for KHE (1). Sirolimus, an mTOR inhibitor acting on the PI3K/AKT/mTOR signaling pathway, has achieved curative effects and a better response in patients with KHE. The mechanism or mechanisms by which sirolimus exerts its effects on KHE may include inhibiting angiogenesis and lymphangiogenesis (17–19). In addition, sirolimus can also inhibit the fibrosis pathway, soften the fibrous area via anti-fibrosis effects, improve the scoliosis caused by local contracture and improve the ROM (20). In the present study, we also demonstrated significant improvements in the associated musculoskeletal complications (e.g., pain and decreased ROM) in patients with spinal KHE after sirolimus treatment. Clinically, surgery can be an approach for tumors when a complete and safe resection can be performed. Some patients with spinal KHE received focal resection. Although there was no evidence of tumor recurrence or metastasis during follow-up, spinal surgery is riskier and not suitable for patients with multiple extensive lesions. In these patients, surgical treatment should not be considered as the first choice. For the KHE patients with KMP, transarterial embolization might work in relieving KMP symptoms as soon as possible (21), but transarterial embolization was more risky in children. For KHE patients whose KMP is difficult to correct with sirolimus, we can try to consider this treatment.

## Conclusion

KHE with spinal involvement is associated with low rate of morbidity. The differential diagnosis and treatment of this disease is sometimes difficult. Current data suggest that sirolimus therapy has a significant effect on the symptoms of KHE in patient with spinal involvement. However, we need more reliable clinical data to verify these findings.

## Data Availability Statement

The raw data supporting the conclusions of this article will be made available by the authors, without undue reservation.

## Ethics Statement

Written informed consent was obtained from the individual(s), and minor(s)' legal guardian/next of kin, for the publication of any potentially identifiable images or data included in this article.

## Author Contributions

TQ and YJ: conception and design. YJ: provision of study material or patients and manuscript revision/review. KY and SD: literature research. TQ and KY: collection and assembly of data. TQ and SD: data analysis and interpretation. SC and TQ: manuscript preparation and manuscript editing. All authors: manuscript final version approval.

## Conflict of Interest

The authors declare that the research was conducted in the absence of any commercial or financial relationships that could be construed as a potential conflict of interest.
